# Clinical significance of tumor-infiltrating lymphocytes and neutrophil-to-lymphocyte ratio in patients with stage III colon cancer who underwent surgery followed by FOLFOX chemotherapy

**DOI:** 10.1038/s41598-019-48140-1

**Published:** 2019-08-12

**Authors:** Yoon Jin Cha, Eun Jung Park, Seung Hyuk Baik, Kang Young Lee, Jeonghyun Kang

**Affiliations:** 1 Department of Pathology, Gangnam Severance Hospital, Yonsei University College of Medicine, Seoul, South Korea; 2 Department of Surgery, Gangnam Severance Hospital, Yonsei University College of Medicine, Seoul, South Korea; 3 Department of Surgery, Severance Hospital, Yonsei University College of Medicine, Seoul, South Korea

**Keywords:** Colon cancer, Tumour biomarkers

## Abstract

Local tumor immune response and host immunity have been suggested as important prognosticators respectively in colorectal cancer. However, the utility of combination of these parameters remains inconclusive. The aim of this study was to investigate the combinational impact of local and host tumor immune response, as determined by tumor-infiltrating lymphocytes (TILs) and neutrophil-to-lymphocyte ratio (NLR), in patients with stage III colon cancer. Patients with stage III colon cancer homogeneously treated with surgery followed by FOLFOX chemotherapy between Jan 2007 and Aug 2013 were included retrospectively. Hematoxylin and eosin (H&E) stained tumor sections of local inflammatory infiltrate (TILs) were classified as 0–3 by the Klintrup-Mäkinen grading method. NLR was measured within 1 month of surgery. The association of NLR and TILs with survival, alone or combined, were measured using multivariate Cox proportional hazard regression analysis. Among 137 patients, 75 (54.7%) were identified as the high TIL group (TILs 2 and 3) and 97 (70.8%) as the low NLR group (NLR < 3). Of the patients with high TILs, 51 (68%) had a low NLR. In univariate analysis, operation time, complications, lymph node ratio (LNR), stage, TILs, and high TILs with low NLR were significantly associated with overall survival(OS). Multivariate Cox regression identified operation time, stage, and TILs as independent risk factors for OS. When high TILs with low NLR vs. others was entered into multivariate analysis, this also proved to be a significant predictor of OS (HR 4.1, 95% CI 1.1–14.2, P = 0.025), with an increased C-index and lower AIC value compared to TILs. Measuring TILs using H&E stained sections could stratify the prognosis of stage III colon cancer. Considering host immunity, using the combination of TILs and NLR, allowed the prognosis to be stratified in more detail.

## Introduction

Colon cancer management includes curative surgical resection and adjuvant chemotherapy if indicated. In stage III colon cancer, adjuvant chemotherapy using the FOLFOX (folinic acid, 5-fluorouracil, and oxaliplatin) regimen is regarded as a standard of care^1^. In a pooled analysis published recently, which tried to compare the noninferiority of 3 months and 6 months adjuvant therapy with either FOLFOX or CAPOX (capecitabine and oxaliplatin), the noninferiority was not confirmed in the overall stage III colon cancer cohort^2^. However, in a lower-risk group, which was defined as T1, T2, or T3 and N1 cancers, it was shown that 3 months of therapy was as effective as 6 months, especially in patients treated with CAPOX. Thus, risk stratification could identify the patients most likely to benefit from more or less chemotherapy treatment and enable clinicians to balance expected survival gain against common therapeutic toxicities such as oxaliplatin-induced neuropathy in patients with stage III colon cancer^3^.

Tumor-infiltrating lymphocytes (TILs) are thought to be an important indicator reflecting the local immune-related tumor microenvironment^4^. An immunoscore has been suggested, which is designed to count tumor-infiltrating T-cells using a combination of both CD3+ and CD8+ densities measured in the invasive margin and tumor center of colon cancer^5^. A recent international validation study has demonstrated that TNM staging and immunoscore remained significant parameters for survival and immunoscore gave the highest relative contribution to the risk of all clinical variables, even including TNM stages in patients with stage I–III colon cancer^6^. However, measuring immunoscore is often time and effort consuming due to additional immunohistochemistry (IHC) staining and equipment requirements. This might be a hurdle in achieving the widespread clinical use of this score. Previously, TILs have been measured using hematoxylin and eosin (H&E) stained tumor sections. Local inflammatory infiltrate was classified as 0–3 using H&E stained tumor sections by the method described by Klintrup-Mäkinen (K-M grading)^7^. It was reported that TILs measured by K-M grading can be used as an important prognostic marker in colorectal cancer^8–10^. Nevertheless, TIL-based stratification when measured by the K-M grading system, has not been thoroughly investigated especially for stage III colon cancer patients who underwent surgery followed by adjuvant FOLFOX chemotherapy.

The role of cellular-mediated inflammatory response in carcinogenesis, which can be represented as lymphocytes, neutrophils, and monocytes in the complete blood cell count, is known to be important^11,12^. The neutrophil-to-lymphocyte ratio (NLR) is a widely used prognostic marker of various tumors including colorectal cancer^13–15^. The relative convenience and non-invasiveness in obtaining NLR is advantageous. Nevertheless, there are still several obstacles on applying NLR in the management of colorectal cancer patients. A very early study investigating the impact of NLR in patients with colorectal cancer revealed that NLR might be dependent on tumor stage and a prognostic impact was not demonstrated in a multivariable analysis^16^. The lack of a generally applicable cut-off value is a main limitation^17^. Although the explanation for the discordance of the results across studies in colorectal cancer is multifactorial, part of the reason may be attributed to the different treatment options especially in the administration of chemotherapy regimens^13^ or simultaneous inclusion of colon and rectal cancer patients. The prognostic impact of NLR on rectal cancer measured before surgery might differ from that of colon cancer because preoperative chemoradiotherapy for indicated rectal cancer patients could change the densities of peripheral inflammatory cells^18^. For these reasons, the prognostic impact of NLR in stage III colon cancer who underwent curative surgery followed by FOLFOX chemotherapy deserves to be investigated, in that the impact of NLR might be analyzed on a cohort who underwent relatively uniform treatments.

Current evidence shows that local immune-related tumor response as well as the host immunity have prognostic value, respectively. As far as we know, few studies considered local and systemic inflammatory response of tumor simultaneously to evaluate their prognostic value^9,19^. Furthermore, whether combination of these two separate immune reactions, although affect each patient’s prognosis at the same time, provide useful prognostic information in stage III colon cancer is unclear. Thus, the aim of this study was to investigate the prognostic impact of TILs and NLR, alone or combined, in patients with stage III colon cancer who underwent surgery followed by FOLFOX chemotherapy.

## Materials and Methods

### Patients

From January 2007 to August 2013, patients who underwent surgery followed by adjuvant FOLFOX chemotherapy for stage III colon cancer at Gangnam Severance Hospital, Yonsei University College of Medicine were initially selected. Patients were included if a slide was available for pathology review and they had undergone a complete blood test within 4 weeks of surgery. Patients who underwent an emergency surgery or palliative surgery (n = 11), who underwent surgery after diagnosed or treated for intestinal obstruction (n = 5), who had inflammatory bowel disease history (n = 2), and who had preoperative chemotherapy (n = 3) were excluded from this study because these factors might influence the systemic or local inflammatory status. Finally, 137 patients were included in our study. All procedures performed in studies involving human participants were in accordance with the ethical standards of the institutional and/or national research committee and with the 1964 Helsinki declaration and its later amendments or comparable ethical standards. This study was approved by the institutional review board (IRB) of Gangnam Severance Hospital. Informed consent was waived for this retrospective study by the IRB.

### Treatments and follow-up

All included patients underwent standard colectomy with regional lymphadenectomy according to the standard surgical principle^20^. All tumor staging was evaluated and classified according to the definition of the 7th American Joint Committee on Cancer and Union for International Cancer Control TNM classification system^21^. Microsatellite instability (MSI) and KRAS status were evaluated according to the techniques described in detail previously^22,23^. However, not all of the patients underwent these two molecular examinations completely. All patients who underwent surgery visited our hospital every 3 months for 3 years and then every 6 months until 5 years. At each follow-up visit, serum carcinoembryonic antigen (CEA) level was measured. Abdominopelvic computed tomography (CT) scans were performed with an average interval of 6 months. Chest CT was done at either 6 or 12-month schedules depending on the discretion of the physician. 18-Fludeoxyglucose (FDG) positron emission tomography (PET) scan was performed on suspicion of recurrence as indicated by the surgeon. Colonoscopy was performed usually 1, 3, and 5 years after surgery. Patient follow-up lasted until the cut-off date (Dec 2017) or the death of the patient. The median follow-up period was 67.8 months (range, 7.5–129.6 months).

### Measurement of TILs and NLR

The patients’ slides were reviewed by an experienced pathologist, who had no information on the clinical outcomes of the patients. TILs were evaluated using slides of H&E stained sections and were scored according to the K-M grading^7^. As described in detail in other studies^8,24^, the deepest area of the invasive margin of the tumor area was assessed by using a 4-degree scale. A score of 0 was given when there was no increase in lymphocytes, 1 denoted a mild and patchy increase in lymphocytes, 2 denoted a moderate and band like lymphocytic infiltrate with some destruction of cancer cell islands, and 3 denoted a marked and florid cuplike lymphocytic infiltrate with frequent destruction of cancer cell islands. Macrophages and granulocytes could be identified under microscope with H&E staining and excluded in TILs measurements. Area of acute inflammation and necrosis was also excluded in measuring TILs. Subsequently, we designated scores 0 and 1 as the low TIL group and scores 2 and 3 as the high TIL group in further analysis.

NLR was measured as the ratio of neutrophils divided by lymphocytes. All included patients underwent a baseline blood test within 30 days of definite surgery. The median number of days from the measurement of NLR to surgery was 6 days (range 1–30 days). The NLR was dichotomized as NLR < 3 (low NLR) and NLR ≥ 3 (high NLR). The cut-off value 3 was derived from previous studies measuring its clinical impact^25,26^.

### Statistical analysis

All statistical analyses were performed using the SPSS software, version 23.0 (IBM SPSS, Chicago, IL, USA) and R package version 3.4.4 (R-project, Institute for Statistics and Mathematics). Differences between groups were analyzed using the chi-square test or Fisher’s exact test for dichotomous parameters. Continuous variables were presented as the mean ± standard deviation and were analyzed using the Student’s *t* test. Overall survival (OS) was determined using the data from the date of surgery until death or last follow-up. Survival curves were constructed using the Kaplan-Meier method and the log-rank test was used to compare survival rates between the groups. In analyses where OS was the outcome, we cross classified TILs and NLR of 3 or greater into four categories (high TILs with low NLR, high TILs with high NLR, low TILs with high NLR, or low TILs with low NLR) and calculated the Kaplan-Meier curves. According to the survival outcomes, the groups were dichotomized as high TILs with low NLR and others, and we used this classification in further statistical analysis.

All variables P < 0.1 on univariate analysis were initially entered into the multivariate analysis. Using multivariable-adjusted Cox proportional hazards regression analysis done by a backward stepwise selection of variables, we evaluated differences in OS by category of TILs and other biomarkers. Models were adjusted for operation time, complications, lymph node ratio (LNR), and stage. Next, we examined NLR and TILs in combination as independent predictors of survival in multivariable-adjusted Cox proportional hazards models. Harrell’s concordance index (C-index) and Akaike information criterion (AIC) were calculated for the comparison of different predictive models. A higher C-index value would indicate better concordance of survival times and a smaller AIC value indicated a more goodness-of-fit for predicting outcomes^27,28^. P < 0.05 was considered to be statistically significant.

## Results

Of 137 stage III colon cancer patients who underwent surgery followed by adjuvant FOLFOX chemotherapy, 13 (9.5%), 49 (35.8%), 44 (32.1%), and 31 (22.6%) patients were classified as K-M grade 0, 1, 2, and 3, respectively. Thus, 75 patients (54.7%) were grouped as high TILs and 62 patients (45.2%) were grouped as low TILs. With regard to NLR, 97 patients (70.8%) and 40 patients (29.1%) showed low NLR (NLR < 3) and high NLR (NLR ≥ 3), respectively. There was greater female predominance in the high TIL group than in the low TIL group (48% in high TILs vs. 25.8% in low TILs, P = 0.008). Tumors larger than 5 cm were significantly more common in the high NLR group than in the low NLR group (P = 0.017). Among patients with high TILs, 51 patients (68%) showed low NLR whereas, 24 patients showed high NLR (P = 0.456) (Table 1, Supplementary data).

**Table 1:** Comparison of patient characteristics and pathologic outcomes according to the TILs and NLR respectively.

Variable		High TILs (n = 75) (%)	Low TILs (n = 62) (%)	*P value*	Low NLR (<3) (n = 97) (%)	High NLR (≥3) (n = 40) (%)	*P value*
Gender	Male	39 (52)	46 (74.2)	0.008	60 (61.9)	25 (62.5)	1.0
	Female	36 (48)	16 (25.8)		37 (38.1)	15 (37.5)	
Age (years)	<70	61 (81.3)	52 (83.9)	0.822	80 (82.5)	33 (82.5)	1.0
	≥70	14 (18.7)	10 (16.1)		17 (17.5)	7 (17.5)	
BMI (kg/m^2^)	<25	58 (77.3)	43 (69.4)	0.332	69 (71.1)	32 (80)	0.299
	≥25	17 (22.7)	19 (30.6)		28 (28.9)	8 (20)	
ASA grade	I	30 (40)	35 (56.5)	0.160	48 (49.5)	17 (42.5)	0.743^*^
	II	36 (48)	21 (33.9)		39 (40.2)	18 (45)	
	III	9 (12)	6 (9.7)		10 (10.3)	5 (12.5)	
CEA (ng/mL)	<5	56 (74.7)	37 (59.7)	0.069	67 (69.1)	26 (65)	0.689
	≥5	19 (25.3)	25 (40.3)		30 (30.9)	14 (35)	
Tumor location^†^	Proximal	25 (33.3)	17 (27.4)	0.465	26 (26.8)	16 (40)	0.155
	Distal	50 (66.7)	45 (72.6)		71 (73.2)	24 (60)	
Operation time (min)	<300	61 (81.3)	49 (79)	0.830	78 (80.4)	32 (80)	1.0
	≥300	14 (18.7)	13 (21)		19 (19.6)	8 (20)	
Complications	No	66 (88)	48 (77.4)	0.113	79 (81.4)	35 (87.5)	0.460
	Yes	9 (12)	14 (22.6)		18 (18.6)	5 (12.5)	
Tumor size (cm)	<5	49 (65.3)	41 (66.1)	1.0	70 (72.2)	20 (50)	0.017
	≥5	26 (34.7)	21 (33.9)		27 (27.8)	20 (50)	
LVI	Negative	48 (64)	33 (53.2)	0.225	55 (56.7)	26 (65)	0.446
	Positive	27 (36)	29 (46.8)		42 (43.3)	14 (35)	
No. of metastatic LNs	Mean ± SD	4.2 ± 4.8	3.9 ± 3.1	0.703	4 ± 4.3	4.2 ± 3.7	0.758
No. of retrieved LNs	Mean ± SD	27.2 ± 14.6	26.7 ± 13.4	0.835	24.8 ± 12.7	32.4 ± 15.7	0.003
LN numbers	<12	3 (4)	4 (6.5)	0.701^*^	7 (7.2)	0	0.106^*^
	≥12	72 (96)	58 (93.5)		90 (92.8)	40 (100)	
LNR	<0.103	39 (52)	29 (46.8)	0.608	47 (48.5)	21 (52.5)	0.710
	≥0.103	36 (48)	33 (53.2)		50 (51.5)	19 (47.5)	
Stage	IIIA	9 (12)	2 (3.2)	0.134^*^	10 (10.3)	1 (2.5)	0.331^*^
	IIIB	48 (64)	40 (64.5)		60 (61.9)	28 (70)	
	IIIC	18 (24)	20 (32.3)		27 (27.8)	11 (27.5)	
MSI	MSS	36 (48)	34 (54.8)	0.237	52 (53.6)	18 (45)	0.439^*^
	MSI-High	4 (5.3)	1 (1.6)		2 (2.1)	3 (7.5)	
	MSI-Low	6 (8)	1 (1.6)		5 (5.2)	2 (5)	
	No data	29 (38.7)	26 (41.9)		38 (39.2)	17 (42.5)	
KRAS	Wild type	19 (25.3)	16 (25.8)	0.299	24 (24.7)	11 (27.5)	0.402
	Mutation	16 (21.3)	7 (11.3)		19 (19.6)	4 (10)	
	No data	40 (53.3)	39 (62.9)		54 (55.7)	25 (62.5)	
High TILs		N/A	N/A		51 (52.6)	24 (60)	0.456
Low TILs		N/A	N/A		46 (47.4)	16 (40)	
NLR < 3		51 (68)	46 (74.2)	0.456	N/A	N/A	
NLR ≥ 3		24 (32)	16 (25.8)		N/A	N/A	

^*^Fisher’s exact test.

^†^Tumor location: Proximal: Cecum – Transverse colon; Distal: Descending colon – Rectosigmoid junction, Two patients with synchronous colon cancer were classified into distal group for statistical reason.

Abbreviations; TILs: Tumor infiltrating lymphocytes; NLR: Neutrophil to lymphocytes ratio; BMI: body mass index; ASA: American society of anesthesiologists; CEA: Carcinoembryonic Antigen; LVI: Lymphovascular invasion; LN: Lymph node; LNR: Lymph node ratio; MSS: Microsatellite stability, MSI: Microsatellite instability.

SD: Standard Deviation.

In univariate analysis, there was a significant difference in 5-year OS between the high TILs and the low TILs (Hazard Ratio, HR: 2.6; confidence interval, CI: 1.1–6.2; P = 0.021). In contrast, there was no survival difference between the low NLR group and the high NLR group (HR: 1.4, CI: 0.6–3.3, P = 0.331). In addition, LNR (P = 0.025), and stage (P = 0.018) were significantly associated with OS. Operation time, complications showed trends for association with OS, but these did not reach statistical significance (Table 2).

**Table 2:** Univariate analysis for overall survival.

		Univariate analysis	
		Hazard Ratio	*P value*
Gender	Male	1	
	Female	1.6 (0.6–3.8)	0.287
Age (years)	<70	1	
	≥70	1.2 (0.4–3.2)	0.701
BMI (kg/m^2^)	<25	1	
	≥25	0.7 (0.2–1.8)	0.477
ASA grade	1	1	0.460
	2	1.6 (0.7–3.6)	0.258
	3	0.8 (0.1–4)	0.881
CEA (ng/mL)	<5	1	
	≥5	0.9 (0.3–2.1)	0.847
Tumor location	Proximal	1	
	Distal	0.9 (0.4–2.2)	0.960
Operation time (min)	<300	1	
	≥300	2.1 (0.9–4.9)	0.08
Complications	No	1	
	Yes	2.2 (0.9–5.2)	0.076
Tumor size (cm)	<5	1	
	≥5	1.5 (0.7–3.4)	0.27
LVI	Negative	1	
	Positive	1.4 (0.6–3.1)	0.368
LN numbers	<12	1	
	≥12	0.6 (0.1–2.9)	0.609
LNR	<0.103	1	
	≥0.103	2.7 (1.1–6.5)	0.025
Stage	IIIC	1	0.018
	IIIB	0.3 (0.1–0.7)	0.007
	IIIA	0.2 (0–1.8)	0.165
TILs	High	1	
	Low	2.6 (1.1–6.2)	0.021
NLR	Low (<3)	1	
	High (≥3)	1.4 (0.6–3.3)	0.331
TILs and NLR	High TILs with low NLR	1	
	Others	4.8 (1.4–16.1)	0.011

Abbreviations; BMI: body mass index; ASA: American society of anesthesiologists; CEA: Carcinoembryonic Antigen; LVI: Lymphovascular invasion; LN: Lymph node; LNR: Lymph node ratio; TILs: Tumor infiltrating lymphocytes; NLR: Neutrophil to lymphocytes ratio.

Tumor location; Proximal: Cecum – Transverse colon; Distal: Descending colon – Rectosigmoid junction, Two patients with synchronous colon cancer were classified into distal group for statistical reason.

**Figure 1:**
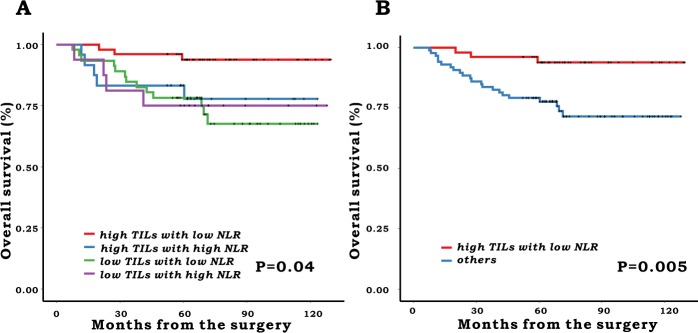
Overall survival according to the combination of TILS and NLR. (**A**) 4 groups comparison (**B**) 2 group comparison.

When we classified patients using the combination of TILs and NLR, there was a significant difference in the 5-year OS between the groups (5-year OS: 93.8% in high TILs with low NLR, 83.3% in high TILs with high NLR, 78.3% in low TILs with low NLR and 75% in low TILs with high NLR, P = 0.04) (Fig. 1A). Thus, we dichotomized patients into two groups: the high TILs with low NLR group versus others. There was also a significant difference in 5-year OS between the newly defined classifications (high TILs with low NLR: 93.8% vs. others: 79.1%, P = 0.005) (Fig. 1B).

Factors with a p-value less than 0.1 in univariate analysis such as operation time, complications, LNR, stage, and TILs were entered into a multivariable analysis in the first stage. Operation time (HR: 2.4, CI: 1–5.6, P = 0.043), stage (IIIC vs. IIIB, HR: 0.3, CI: 0.1–0.7, P = 0.009), and TILs (HR: 2.5, CI: 1–5.9, P = 0.032) were proved to be independent risk factors for OS (Model 1). In the second stage, we entered high TILs with low NLR versus others, instead of TILs, into a multivariable model (Model 2). The results showed that stage (IIIC vs. IIIB, HR: 0.3, CI: 0.1–0.7, P = 0.007), and high TILs with low NLR (HR: 4.1, CI: 1.1–4.2, P = 0.025) were independently associated with OS. Model 2 showed higher fitness in terms of Akaike information criterion (AIC) (226.289 in Model 2 vs. 229.221 in Model 1) and higher predictive power in terms of C-index [0.737, 95% CI (0.623–0.850) in Model 2 vs. 0.717, 95% CI (0.604–0.831) in Model 1] than Model 1 (Table 3).

**Table 3:** Multivariate analysis using different parameters.

		Model1		Model 2	
		Hazard Ratio (CI)	*P value*	Hazard Ratio (CI)	*P value*
Operation time (min)	<300 vs. ≥300	2.4 (1–5.6)	0.043		
Complications	No vs. Yes			2.2 (0.9–5.4)	0.075
LNR	<0.103 vs. ≥0.103				
Stage	IIIC	1	0.026	1	0.023
	IIIB	0.3 (0.1–0.7)	0.009	0.3 (0.1–0.7)	0.007
	IIIA	0.2 (0–2.3)	0.243	0.3 (0–2.8)	0.325
TILs	High vs. Low	2.5 (1–5.9)	0.032		
TILs and NLR	High TILs and low NLR vs. others			4.1 (1.1–14.2)	0.025
C-index	(95% Confidence Interval)	0.717(0.604–0.831)		0.737(0.623–0.850)	
AIC		229.221		226.289	

Abbreviations; LNR: Lymph node ratio; TILs: Tumor infiltrating lymphocytes; NLR: Neutrophil to lymphocytes ratio.

C-index: Harrell’s concordance index; AIC: Akaike information criterion.

Factors with p value less than 0.1 in univariate analysis were entered into multivariate analysis.

**Figure 2:**
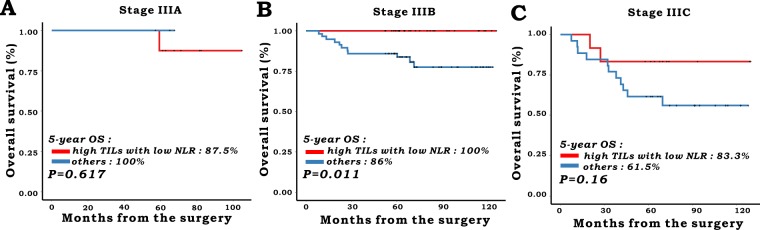
Overall survival according to the combination of TILs and NLR in each stage. (**A**) Stage IIIA (n = 11), (**B**) Stage IIIB (n = 88), (**C**) Stage IIIC (n = 38).

In subgroup analysis according to each stage, 11, 88, and 38 patients were classified into stage IIIA, IIIB, and IIIC, respectively. Patients with high TILs with low NLR showed better 5-year OS in the stage IIIB group (100% in high TILs with low NLR group vs. 86% in others, P = 0.011). In contrast, there was no difference between the two groups in stage IIIA (P = 0.617) and stage IIIC (P = 0.16), respectively (Fig. 2).

## Discussion

This study has demonstrated that TILs measured by H&E staining could stratify the prognosis of patients with stage III colon cancer who underwent surgery followed by FOLFOX chemotherapy. Although NLR per se could not predict prognosis in our cohort, a combination of TILs and NLR could allow us to distinguish the survival outcomes of patients in more detail. Thus, considering local tumor response and systemic immunity by TILs measured by H&E staining and NLR might be an affordable and effective option for risk stratification in patient with stage III colon cancer.

The clinical impact of IHC-based measurements of TILs such as CD3, CD8, CD45, and FOXP3 have been widely investigated in colorectal cancer^29–34^. According to a recent international validation study, immunoscore, measuring total tumor-infiltrating and cytotoxic tumor-infiltrating T-cells, can stratify patients’ outcomes more definitely than TNM stage^6^. However, one concern of this approach is that a considerable number of patients (858/3539, 24%) in the retrospective study were filtered by quality control and could not be included in their multinational study^6^. Among them, 41.6% (357/858) of the excluded patients were associated with the failure of obtaining proper IHC results. The IHC-based measurement may not be always successful in some environments with scarce resources, which prevents the widespread adoption of IHC. Many studies have evaluated the impact of TILs measured by the H&E staining of sections from colorectal cancer patients. Huh *et al*. analyzed 546 colorectal cancer patients and concluded that TILs have a prognostic impact^8^. Interestingly, the impact of TILs was demonstrated only in stage III colorectal cancer, and not in stage I and II colorectal cancer patients. In that study, however, various chemotherapy agents were used, and FOLFOX chemotherapy, which is regarded as the standard chemotherapy for stage III colon cancer in current practice, was used for only 6 patients (1%). Another study measured the TILs of colorectal cancer patients according to the recommendation of the International TILs Working Group in breast cancer^10^. The patients were composed of stage II and stage III colon and rectal cancer patients with or without adjuvant chemotherapy^10^. In their study, the density of TILs, where high TIL level was defined as more than 42%, was independently associated with OS in multivariate analysis. Although TILs were proven to be a significant predictor of survival, the results were derived from a heterogeneous cohort, which included stage II and III rectal cancer patients who did not receive radiation therapy. In addition, TILs measured by H&E staining in colorectal cancer have been thoroughly investigated by the one group and these studies demonstrated that local tumor infiltrate was associated with survival^24,35,36^. Nevertheless, one concern might be that their studies showed a relatively low incidence of 12 or more lymph nodes examined^24,35,36^. Thus, it was difficult to completely exclude the possibility that surgical quality affected the survival outcomes. Therefore, these previous studies cannot definitively inform us that the TILs defined by H&E stain may have an impact on the prognosis of patients with stage III colon cancer who underwent FOLFOX chemotherapy. Our study demonstrated that TILs can be used as an independent prognostic factor for patients with stage III colon cancer. The strength of the present study is that our group included relatively homogeneously treated patients with a high proportion of adequately retrieved lymph nodes (95%) and all patients had undergone adjuvant FOLFOX chemotherapy. Our result can be applied relatively easily in clinical practice because an H&E examination is a routine procedure for the evaluation of tumor staging.

The clinical impact of NLR per se was not demonstrated in our study. Although many studies concluded that NLR is an important inflammatory biomarker in colorectal cancer, there are several issues to be mentioned. As depicted in one of the earlier studies, which investigated the impact of NLR in colorectal cancer, Walsh and colleagues showed that an NLR greater than 5 correlated with OS only in univariate analysis^16^. Because NLR was dependent on Dukes stage, the significance was lost in multivariate analysis^16^. According to a study by Li and colleagues, which included 5,336 patients and is one of the largest scale studies dealing with this issue for colorectal cancer, NLR dichotomized as 2.72 can predict patients’ OS^37^. However, in that study, the clinical impact of inflammatory markers including NLR was not evident in patients who did not undergo adjuvant chemotherapy. The authors explained that the significance of NLR might not be demonstrated due to the overall good survival outcome of this subgroup. Malietzis and colleagues analyzed the impact of NLR in 506 colorectal cancer patients who did not undergo adjuvant chemotherapy^25^. They showed that an NLR of more than 3 was identified as an independent prognostic factor for disease-free survival. Interestingly, this correlation did not last when OS was evaluated. Although we could not elucidate the reason of the negative impact of NLR per se in our group, it is worth mentioning that most of the previous studies evaluating the impact of NLR in colorectal cancer include heterogeneous groups of patients. Most of the studies included colon cancer and rectal cancer patients simultaneously and patients who either underwent adjuvant chemotherapy or did not. In addition, the chemotherapeutic agents and regimens were diverse and included 5FU, capecitabine, FOLFOX, etc. As we already know, rectal cancer patients usually underwent preoperative or postoperative radiation therapies, thus the radiation effect cannot be ignored in the statistical analysis. In node-positive colon cancer patients, the benefit of adjuvant chemotherapy is well established. Thus, the real effects of NLR might be more fairly evaluated in environments where chemotherapy was considered as a treatment option. Nevertheless, considering our relatively small number of patients, whether NLR has an impact on patients with stage III colon cancer remains undetermined.

Pine *et al*. analyzed the correlation of TILs and NLR and its impact on survival using 358 colorectal cancer patients^19^. In their study, TILs (expressed as a lymphocytic reaction at the invasive margin in their paper) were correlated with the NLR, which was divided using a cut-off value of 5. There was a significantly lower rate of patients with more than 5 NLR in the high TIL group (15.3% in the high TIL group vs. 29.2% in the low TIL group, P = 0.005). NLR dichotomized as 5 was proven to be an independent prognostic factor for OS, however, TILs were not an independent factor in multivariate analysis. The authors did not include the combination of these two parameters in statistical analysis. Contrary to their study, our study showed no direct correlation between TILs and NLR, however, TILs have an impact on OS and NLR did not. Again, we want to point out there were some differences in the inclusion criteria and OS outcomes might differ between the two studies because of the difference of included study periods.

One of the interesting findings in our study is that the combination of TILs with NLR had a higher relative contribution to the risk stratification of patients’ survival than TILs alone. Patients with high TILs showed different prognosis according to the NLR group (5-year OS; high TILs with low NLR, 93.8%, vs. high TILs with high NLR, 83.3%, P = 0.038). In contrast, this kind of discrimination was not demonstrated in patients in the low TIL group (5-year OS; low TILs with low NLR, 78.3%, vs. low TILs with high NLR, 75%, P = 0.892). Although the underlying reason for this association is not evident, it has been speculated that the NLR might have a partial effect on patient survival. One step further, our study showed that even among the same sub-staging, risk stratification may be possible according to the combination of local tumor immune response and host immunity defined as TILs and NLR. In patients with stage IIIB, the OS was significantly better in patients with high TILs with low NLR than in others (P = 0.011), although this correlation was not demonstrated in patients with stage IIIA or IIIC. Considering the distinct difference between groups of 5-year OS in stage IIIC (83.3% vs. 61.5%, P = 0.16) and the low number of included patients with stage IIIA (n = 11), these results might be derived from a type II error, which might be a limitation of this retrospective study. Further study is warranted to validate our findings.

This study has several limitations, which deserve to be mentioned. The small number of patients included in this retrospective study might be a main limitation. The TIL measurement was performed by one single pathologist, which might be a source of bias. However, previous studies evaluating inter-observer agreement on this subject showed good agreement (kappa value: 0.81 in each study)^24,35^. The cut-off value to discriminate between the high or low group using NLR is not strongly established. According to a recent review^13^, the cut-off value ranged from 2 to 5 and the proportion of the high NLR group might be different between the studies or may be diverse between races. This might be a critical limitation in the general application of this value^17^. Although our group used 3 as a reference value for dichotomization, if we applied the cut-off value as 5 or 2.72 (which was recommended by Li and colleagues^37^), there was no difference of OS between the two subgroups divided by NLR, respectively. There are several reports on the clinical impact of MSI, KRAS, NRAS, and BRAF mutations in the prognosis of colorectal cancer^38–40^. The effect of these genomic alterations in colorectal cancer may enter into the multivariable analysis; however, due to the retrospective study design, it was not possible to include all these variables in the analysis. Finally, the H&E-based K-M grading of inflammatory infiltrate has some inherent limitations compared to the IHC-based measurements. As explained in several previous studies^6,36^, the K-M grade provides a measure of the overall, generalized inflammatory cell infiltrate, in contrast, immunoscore measured the host adaptive T-lymphocyte. Consequently, the K-M grade can be elevated by not only the increasing tumor-infiltrating T lymphocytes, but also by increasing the density of the innate immune infiltrate such as neutrophils and macrophages^6,36^. Improvement of prognostic power by the combination of TILs and NLR in our study might suggest one possibility to overcome this limitation of K-M based tumor infiltrate measurements, although these results should be validated in other cohorts. In fact, Turner and colleagues have already demonstrated that the combination of two markers, such as intratumoral immune infiltrate and NLR, could further stratify the prognosis independent of standard high-risk criteria. Patients with prominent systemic and local inflammatory response (low chronic inflammatory cell density with high NLR) showed the worst outcome (5-year OS 55.8%) in stage II colon cancer^9^.

In conclusion, our study demonstrated that TILs measured by H&E staining and a combination of TILs and NLR could stratify patients’ survival in stage III colon cancers. These parameters can be obtained during clinical practice without any additional effort or equipment. Thus, we believe this approach is worth considering in view of cost-effectiveness and convenience. Evaluation of chemotherapy efficacy according to risk stratification using these easily obtained biomarkers may be required to select patients who may benefit from reduced chemotherapy in stage III colon cancer.

## Supplementary information

Supplementary Dataset 1
