# Supplementary material for: Clinical significance of tumor-infiltrating lymphocytes and neutrophil-to-lymphocyte ratio in patients with stage III colon cancer who underwent surgery followed by FOLFOX chemotherapy
Source: Sci Rep. 2019 Aug 12;9:11617. doi: 10.1038/s41598-019-48140-1 (PMC6690947; doi:10.1038/s41598-019-48140-1)
Supplement: Supplementary file 1 — Supplementary Dataset 1 [file 41598_2019_48140_MOESM1_ESM.docx]

**Supplementary data**

Clinical significance of tumor-infiltrating lymphocytes and neutrophil-to-lymphocyte ratio in patients with stage III colon cancer who underwent surgery followed by FOLFOX chemotherapy

Yoon Jin Cha, M.D., Ph.D.^1^, Eun Jung Park, M.D., Ph.D.^2^, Seung Hyuk Baik, M.D., Ph.D.^2^, Kang Young Lee, M.D., Ph.D.^3^, Jeonghyun Kang, M.D., Ph.D.^2^

Fig S1. Histogram and distribution of NLR


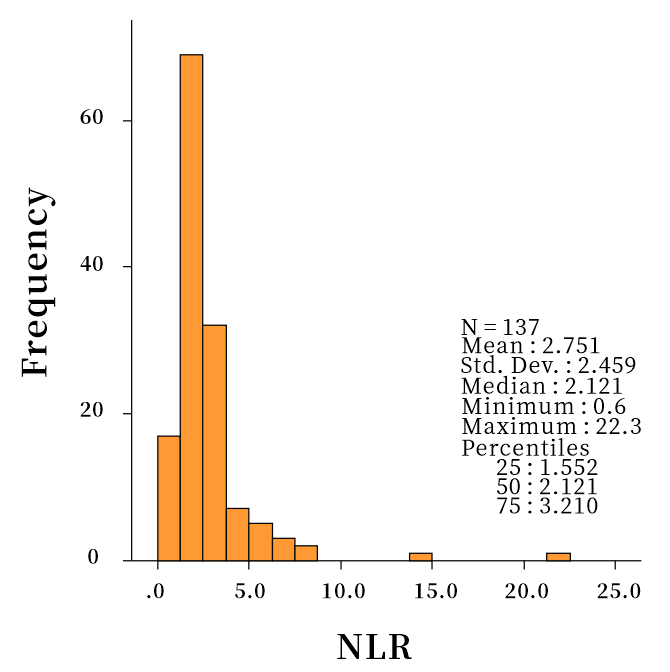


Fig S2. Distribution of NLR according to the TILs grading

| A) NLR according to TILs grade 0 - 4 | B) NLR according to the low and high TILs |
| --- | --- |
| 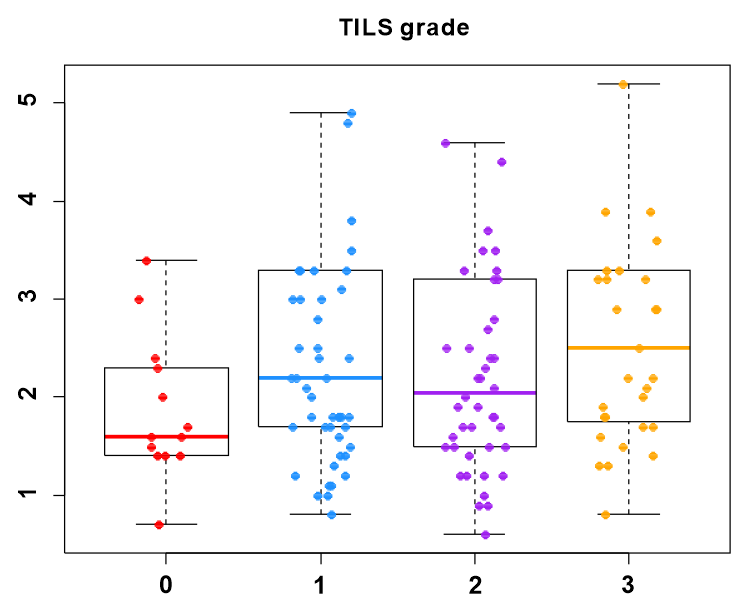 | 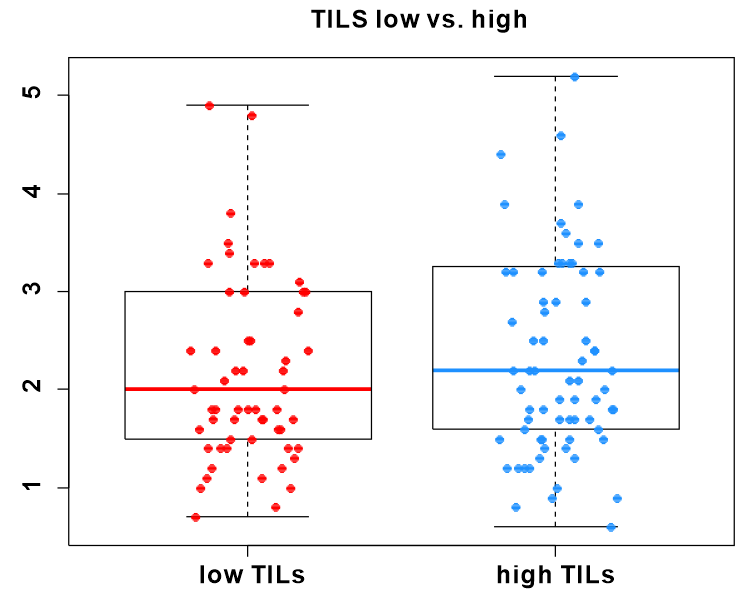 |
| p = 0.1703 (Kruskal-Wallis test) | p = 0.4553 (Mann–Whitney *U* test) |

Fig S3. Distribution of NLR according to the clinicopathological parameters

| A) NLR according to the gender | B) NLR according to the age |
| --- | --- |
| 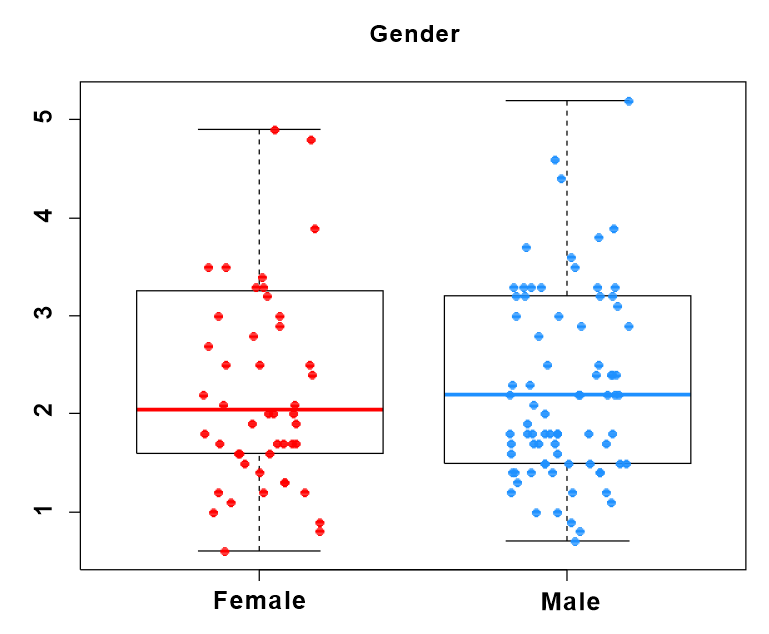 | 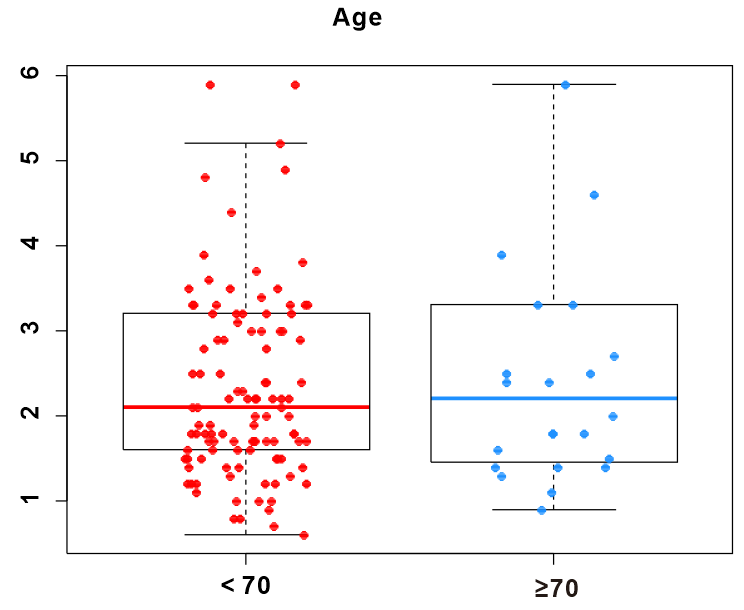 |
| p = 0.977 (Mann–Whitney *U* test) | p = 0.894 (Mann–Whitney *U* test) |
|  |  |
| C) NLR according to the BMI | D) NLR according to the ASA grade |
| 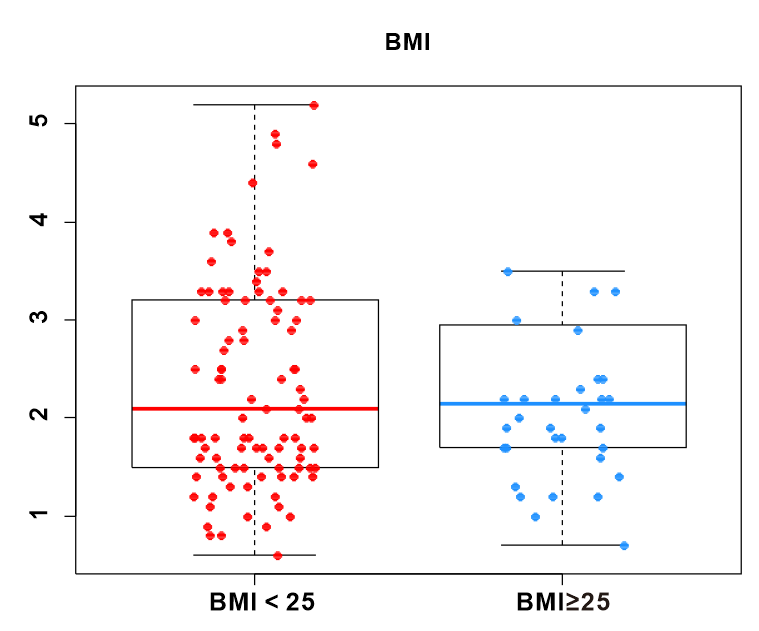 | 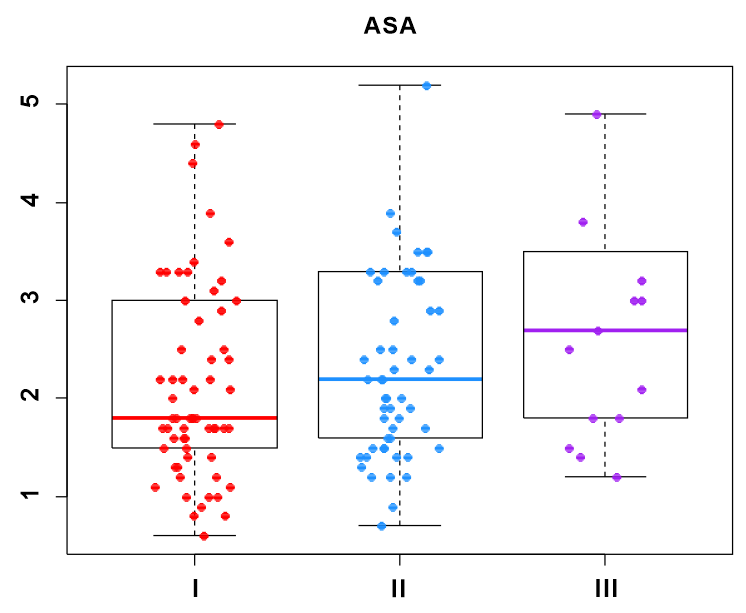 |
| p = 0.9766 (Mann–Whitney *U* test) | p = 0.1958 (Kruskal-Wallis test) |
|  |  |
| E) NLR according to the CEA | F) NLR according to the Tumor location |
| 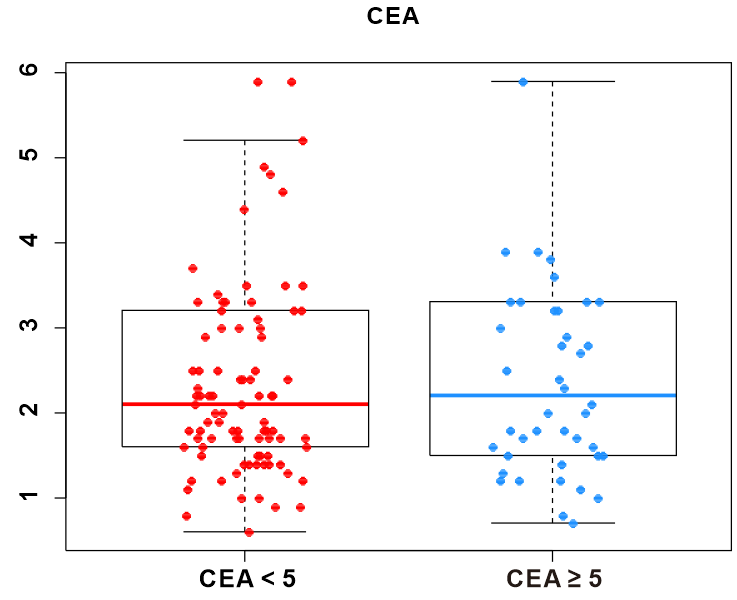 | 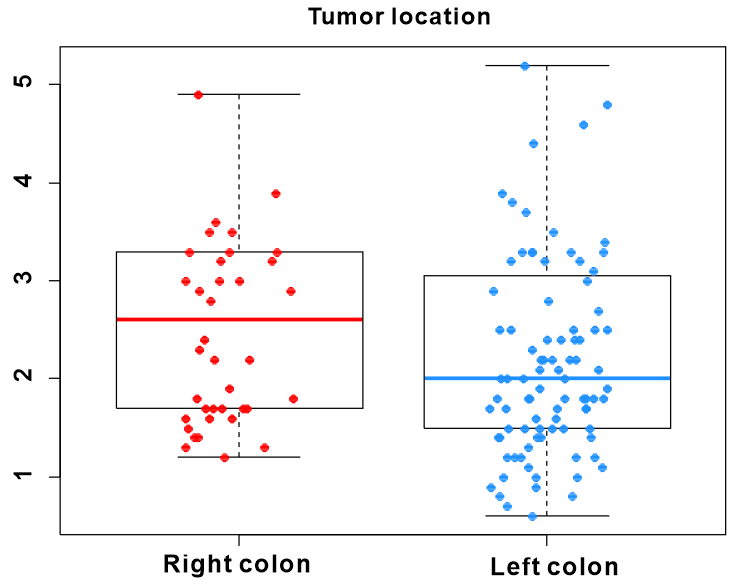 |
| p = 0.8572 (Mann–Whitney *U* test) | p = 0.0709 (Mann–Whitney *U* test) |
|  |  |
| G) NLR according to the complication | H) NLR according to the Tumor size |
| 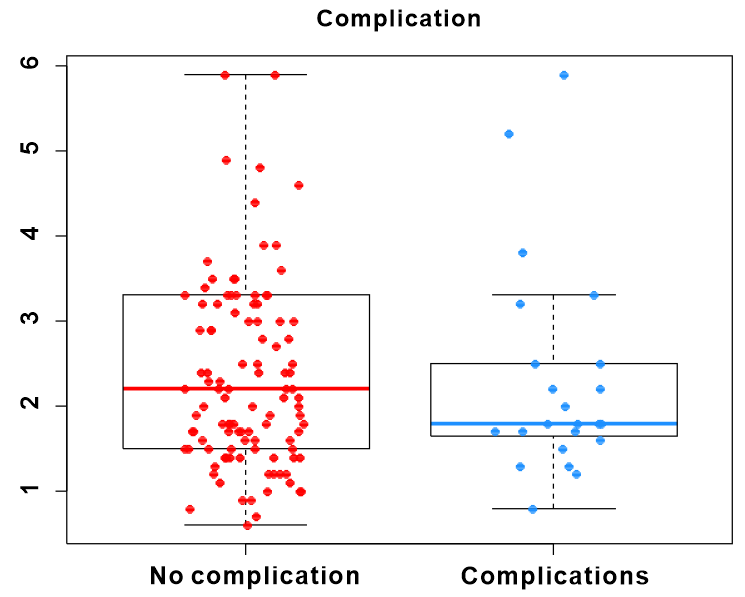 | 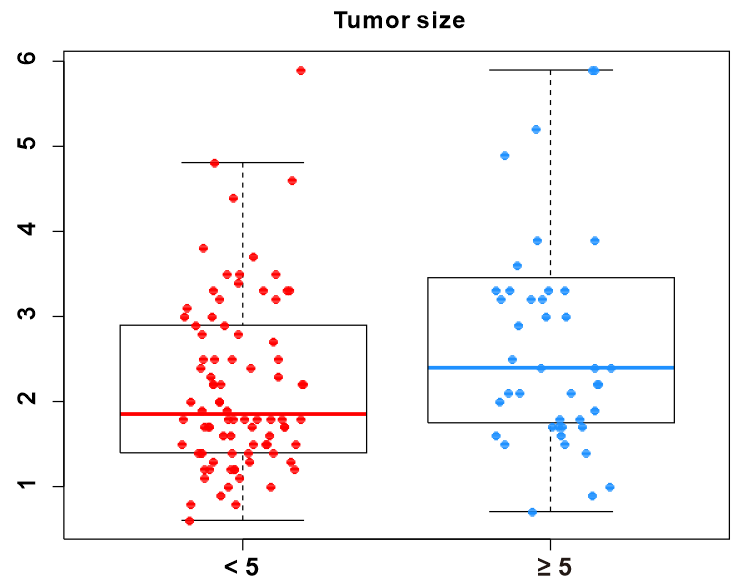 |
| p = 0.7033 (Mann–Whitney *U* test) | p = 0.008 (Mann–Whitney *U* test) |
|  |  |
| I) NLR according to the LVI | J) NLR according to the Stage |
| 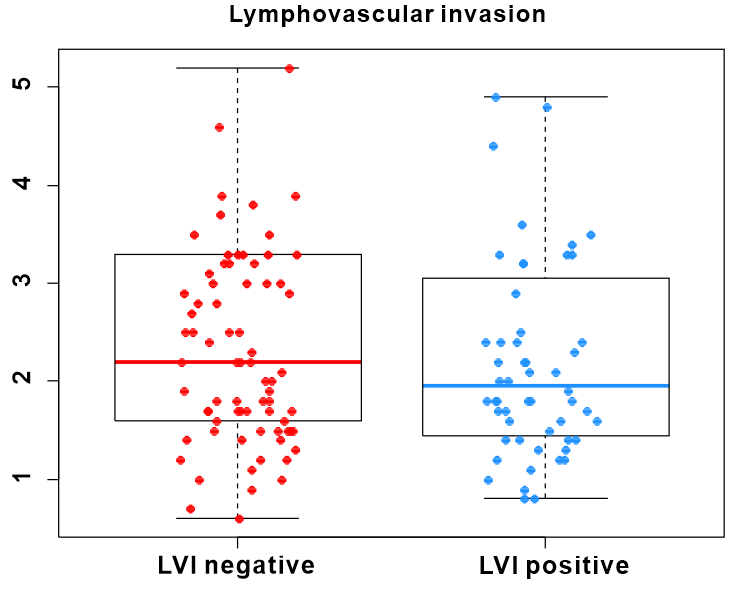 | 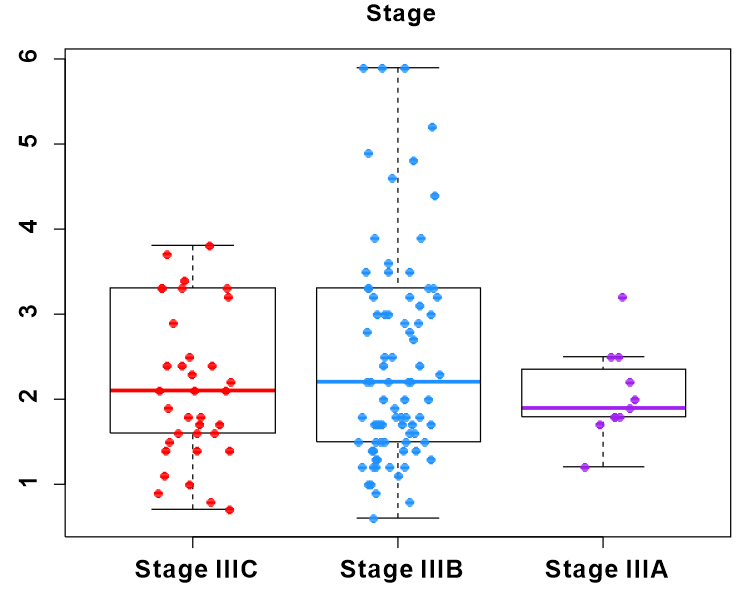 |
| p = 0.2273 (Mann–Whitney *U* test) | p = 0.8314 (Kruskal-Wallis test) |
|  |  |
| K) NLR according to the MSI status | L) NLR according to the KRAS mutation status |
| 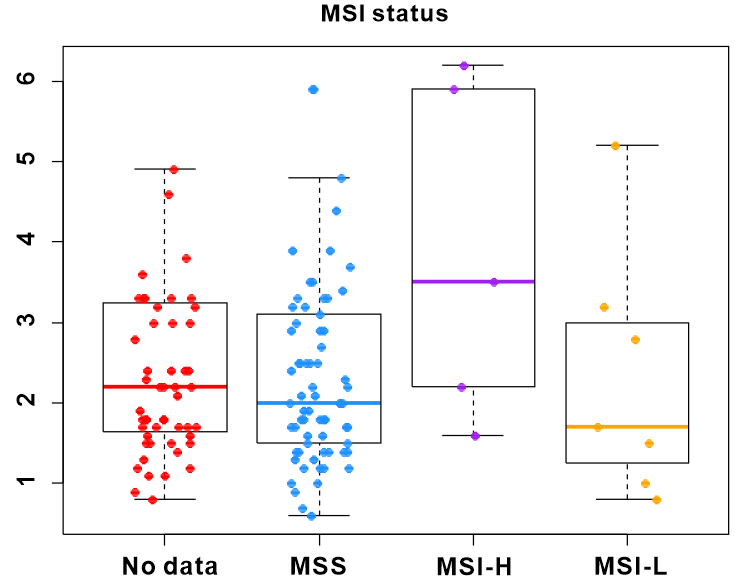 | 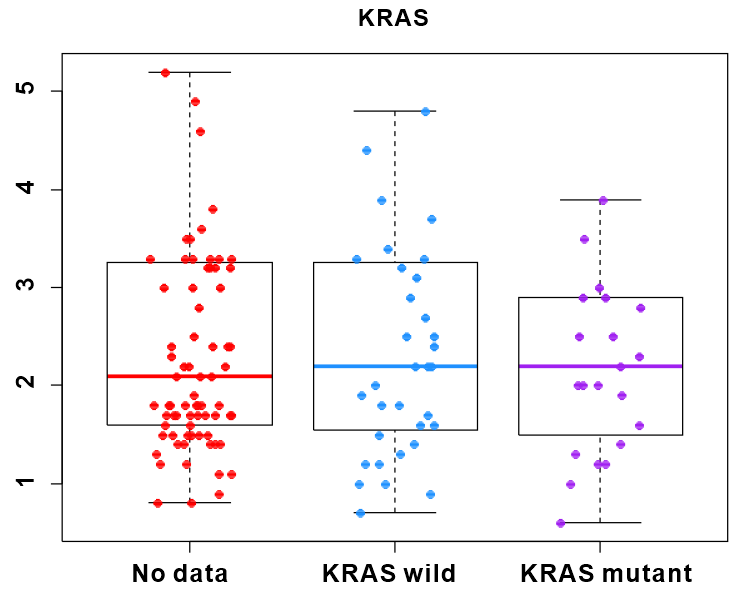 |
| p = 0.3467 (Kruskal-Wallis test) | p = 0.9939 (Kruskal-Wallis test) |

Fig S4. Kaplan Meier plots for overall survival according to the different cut-offs based on the NLR

| A) Mean | B) Median |
| --- | --- |
| 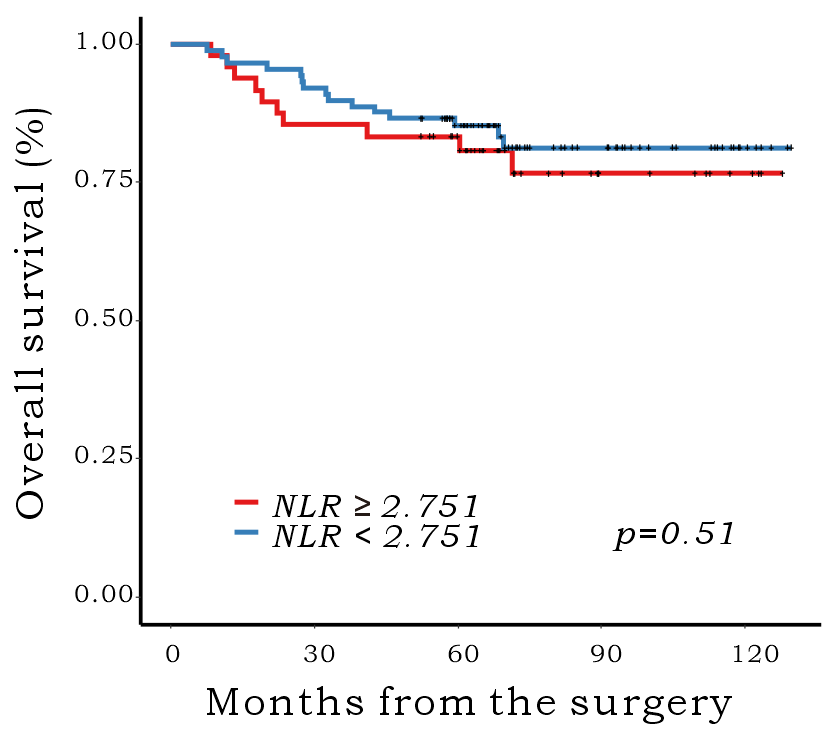 | 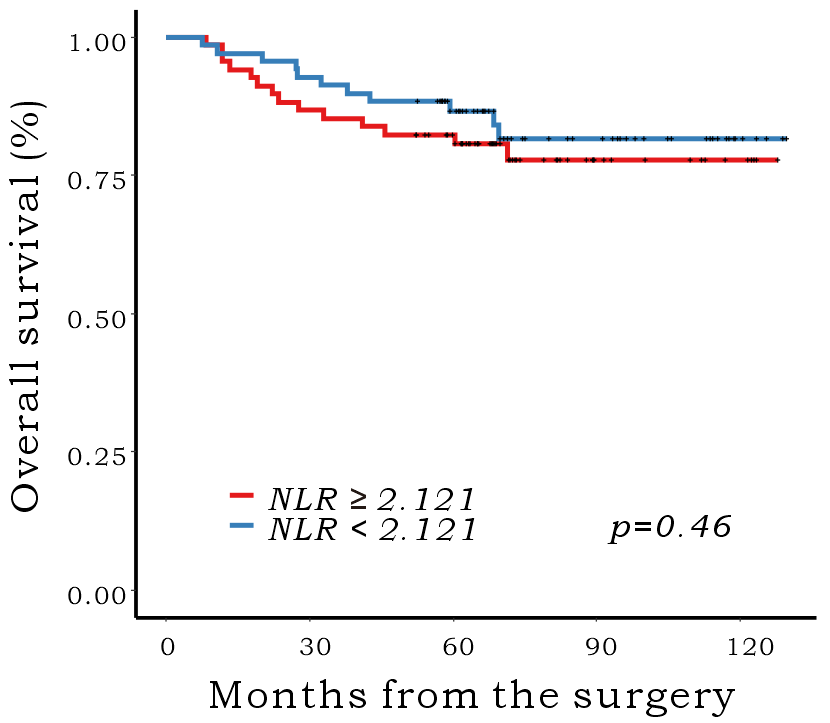 |
|  |  |
| C) NLR according to the 25 percentile | D) NLR according to the 75 percentile |
| 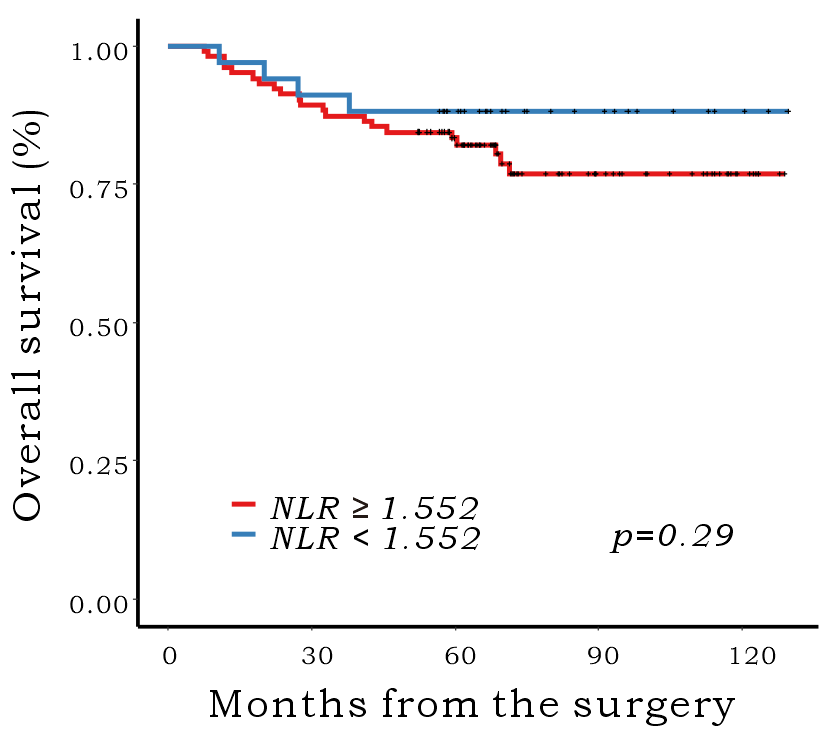 | 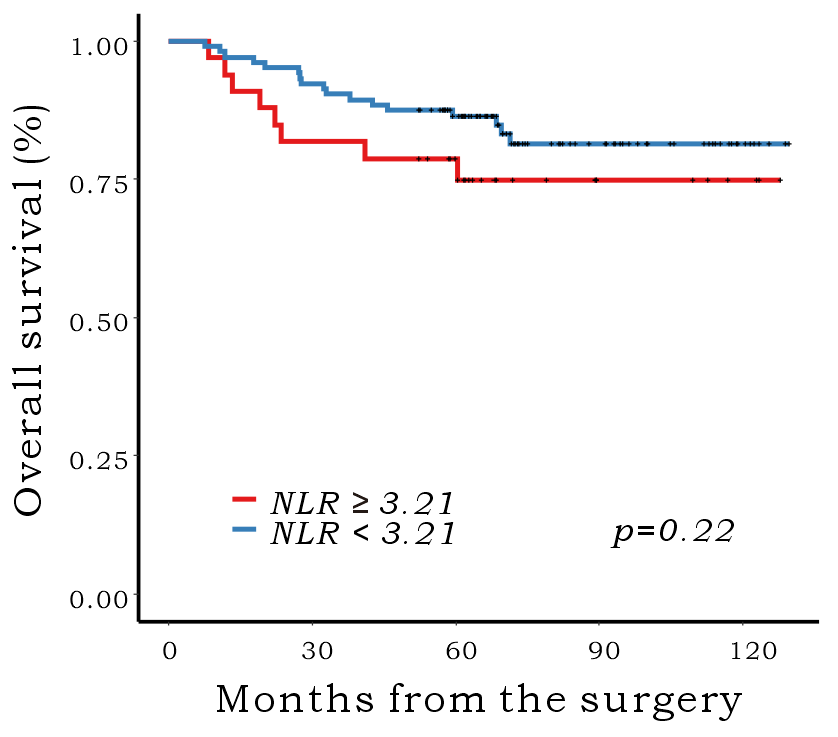 |

Fig S5. Determining cut-off value to maximize the survival difference and Kaplan Meier plot according to this value

: Optimal stratification is based on log-rank statistics to test for a threshold value of a continuous variable with respect to overall survival.

| A) Freqency based on the cut-off | B) Kaplan Meier Plot according to the cut-off |
| --- | --- |
| 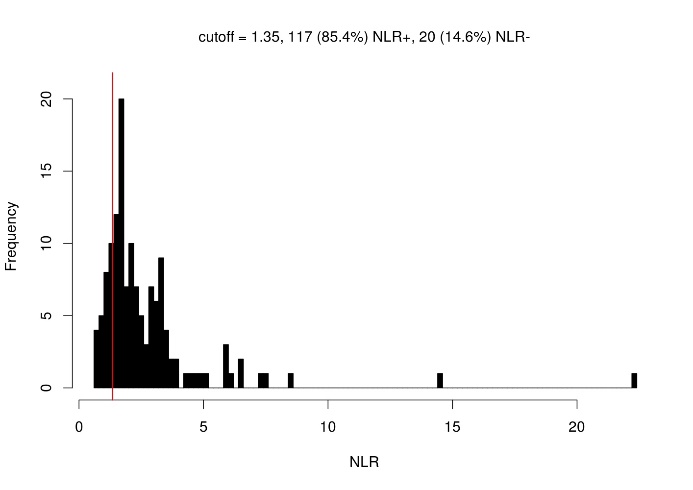 | 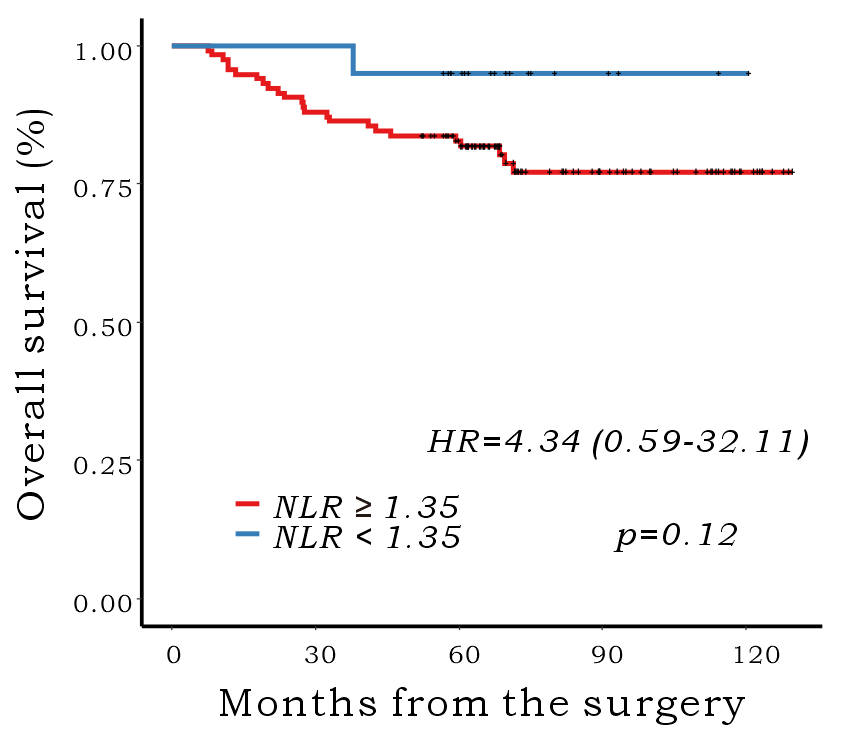 |

Table S1. Frequencies of neutrophil and lymphocyte

|  |  | Neutrophil (10^3^/㎕) | Lymphocyte (10^3^/㎕) |
| --- | --- | --- | --- |
| Mean |  | 4.26 | 1.81 |
| Median |  | 3.84 | 1.7 |
| Std. Deviation | | 2.20 | 0.75 |
| Minimum |  | 1.68 | 0.52 |
| Maximum | | 14.9 | 7.1 |
| Percentiles | 25 | 2.87 | 1.35 |
|  | 50 | 3.84 | 1.7 |
|  | 75 | 4.94 | 2.17 |

Fig S6. Distribution of Neutrophil and lymphocyte according to the TILs grading

| A) Neutrophil according to TILs grade 0 - 4 | B) Neutrophil according to the low and high TILs |
| --- | --- |
| 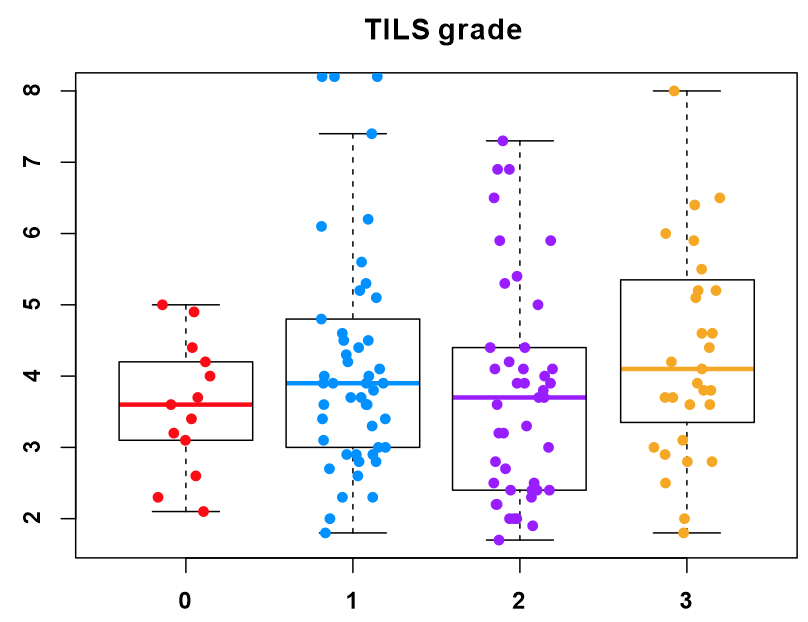 | 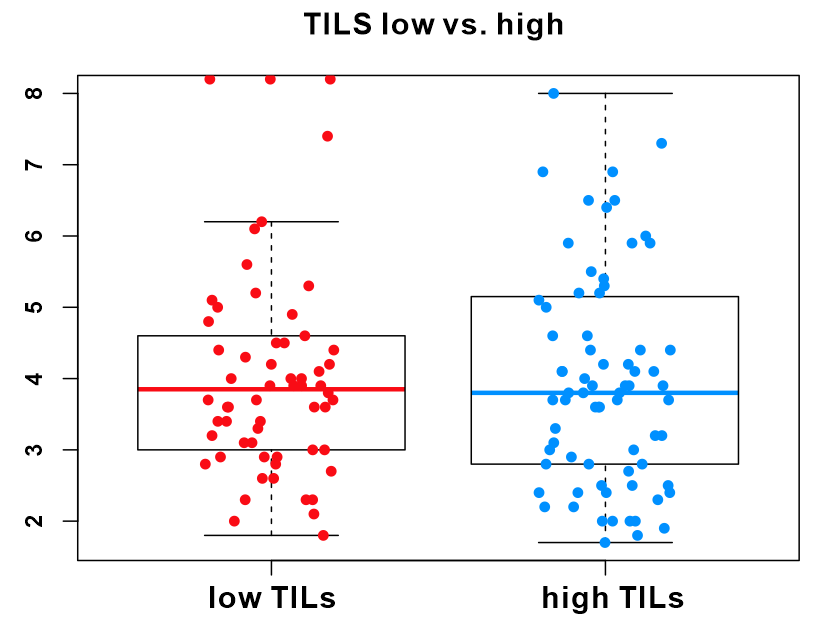 |
| p = 0.1977 (Kruskal-Wallis test) | p = 0.8271 (Mann–Whitney *U* test) |
| C) Lymphocyte according to TILs grade 0 - 4 | D) Lymphocyte according to the low and high TILs |
| 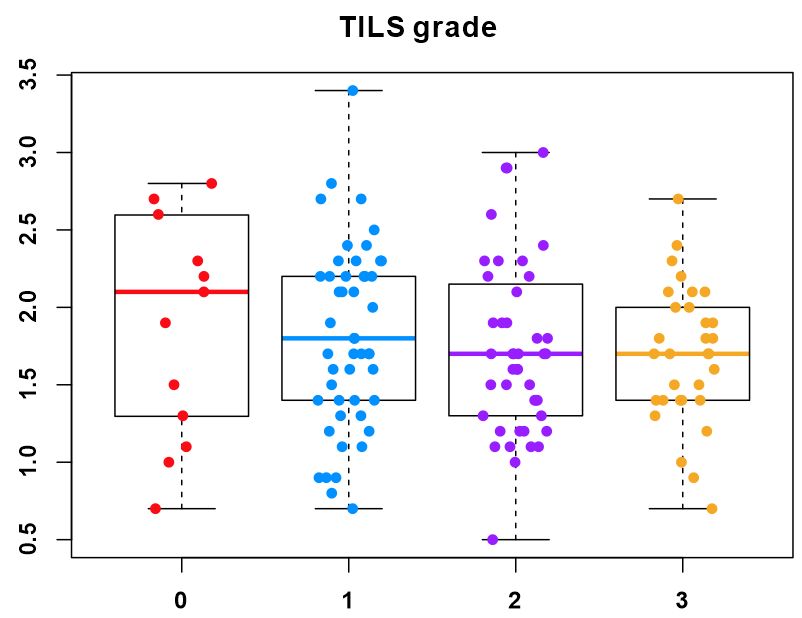 | 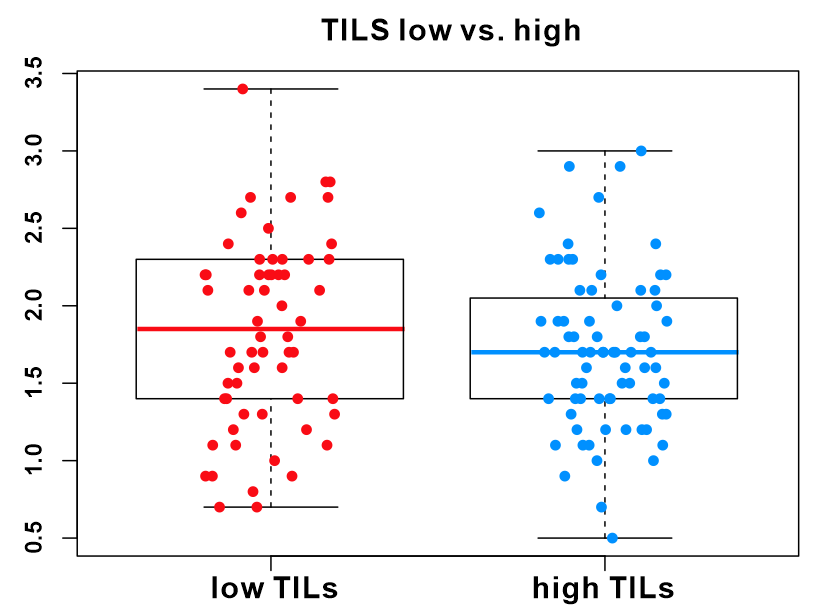 |
| p = 0.5883 (Kruskal-Wallis test) | p = 0.1922 (Mann–Whitney *U* test) |
|  |  |

Fig S7. Kaplan Meier plots for overall survival according to the different cut-offs based on the neutrophil and lymphocyte

| A) Neutrophil - mean | B) Neutrophil - median |
| --- | --- |
| 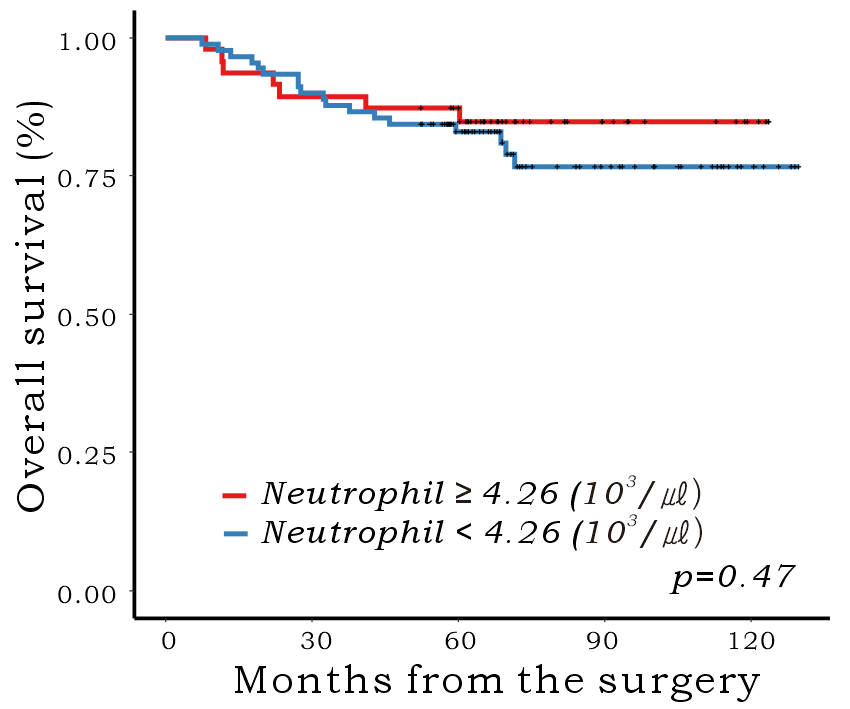 | 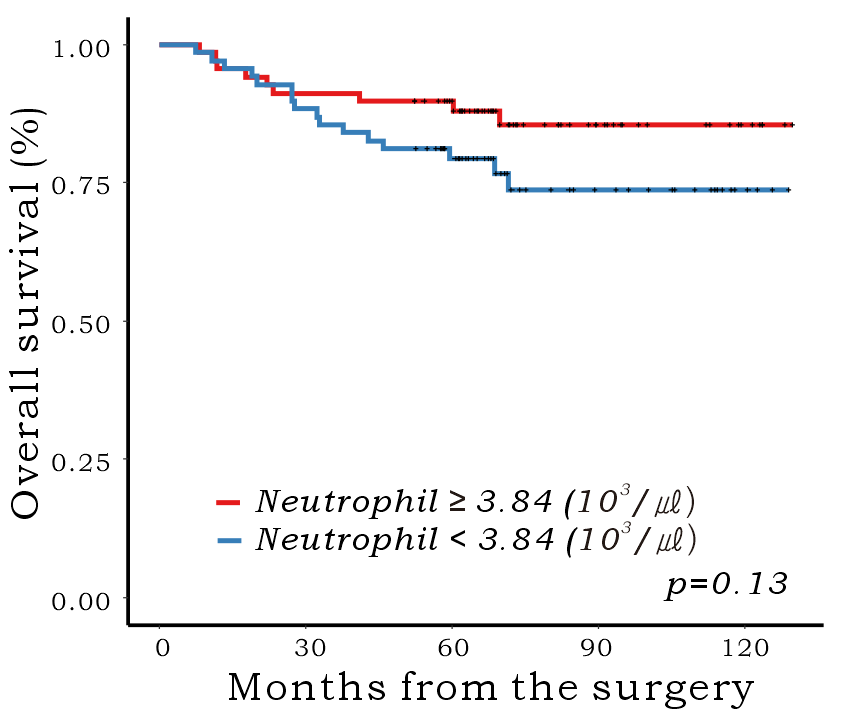 |
| C) Lymphocyte - mean | D) Lymphocyte - median |
| 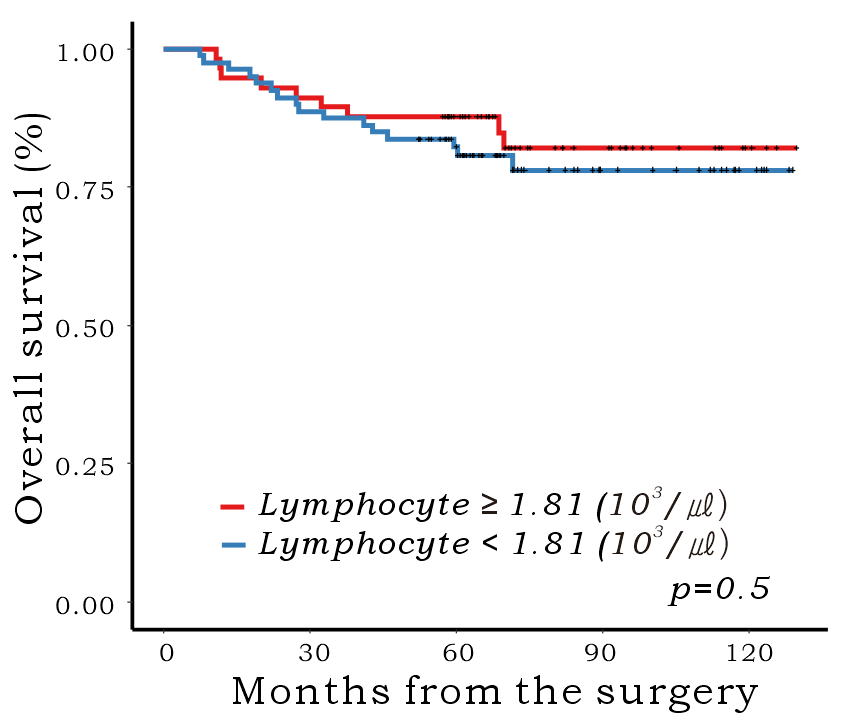 | 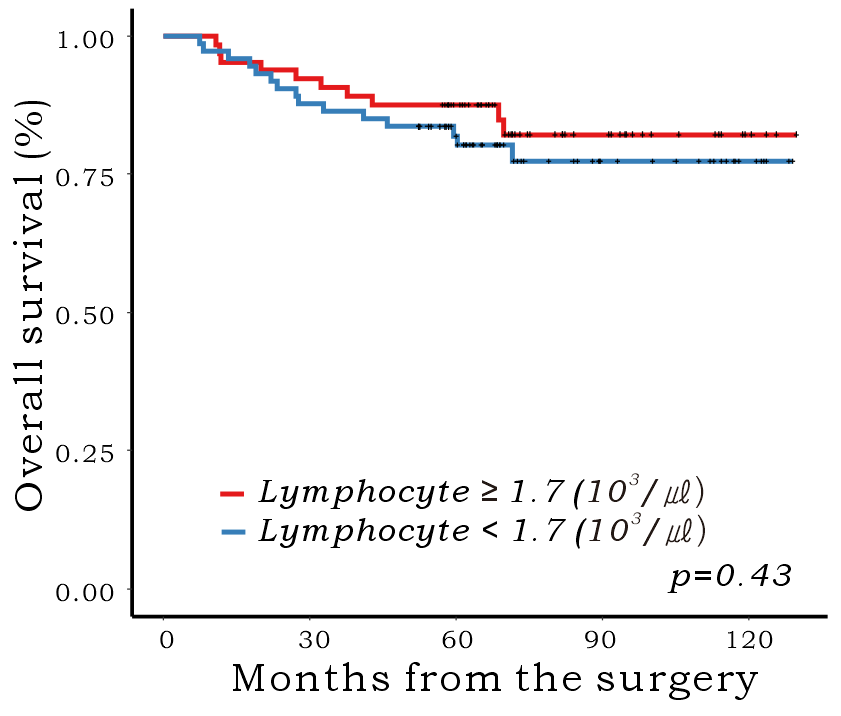 |

Table S2. Univariate analysis of neutrophil and lymphocyte counts for overall survival

| Univariate analysis | | | |
| --- | --- | --- | --- |
|  |  | Hazard Ratio | *P value* |
| Neutrophil (10^3^/㎕) | ≥ 3.84 | 1 |  |
|  | < 3.84 | 1.9 (0.8 – 4.3) | 0.118 |
| Lymphocyte (10^3^/㎕) | ≥ 1.7 | 1 |  |
|  | < 1.7 | 1.25 (0.5 – 2.7) | 0.578 |

Fig S8. Scatter plot correlations between continuous variables

| A) NLR vs. Age | B) NLR vs. BMI |
| --- | --- |
| 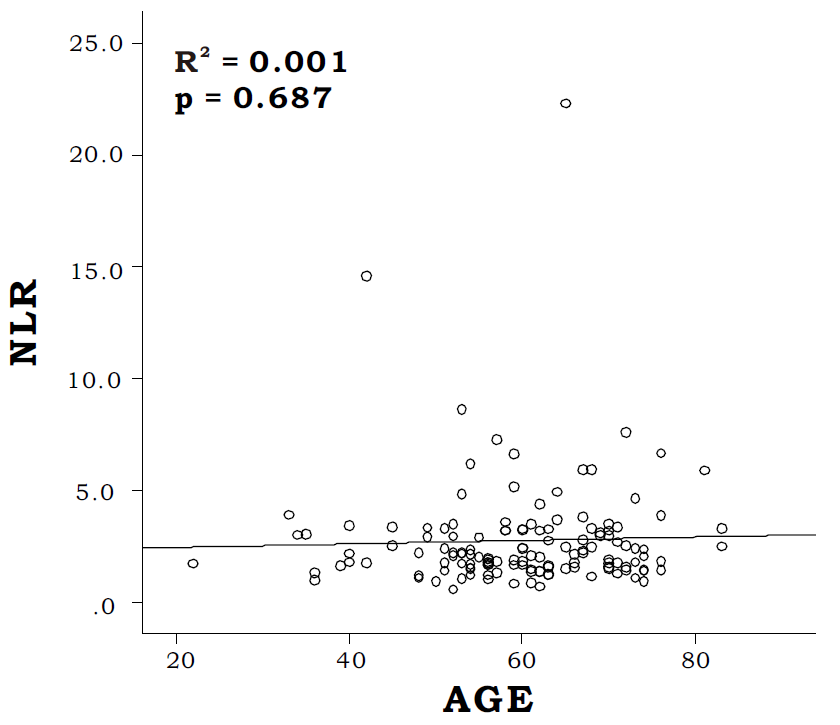 | 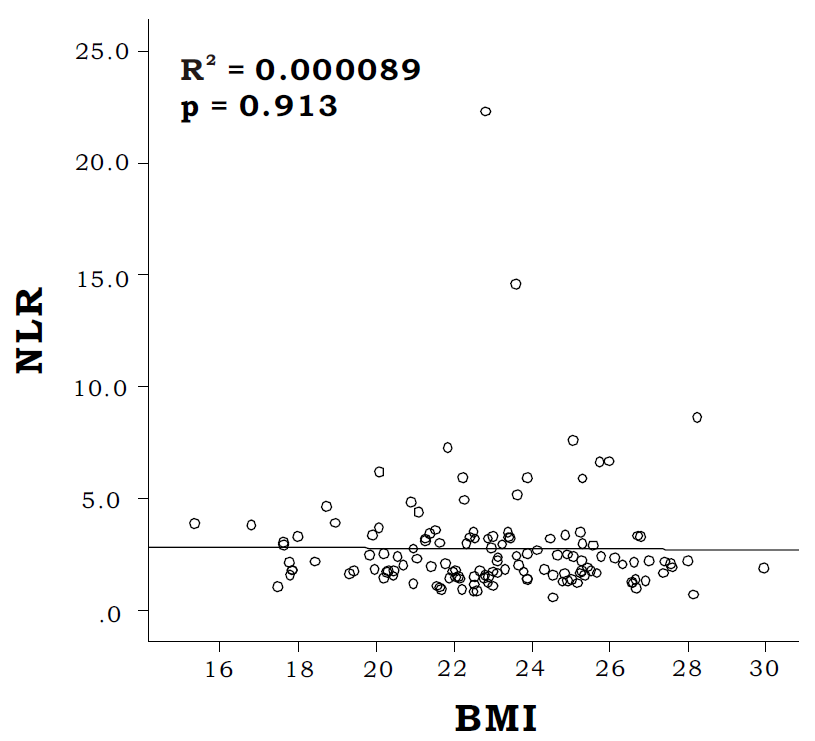 |
| C) Neutrophil vs. Age | D) Neutrophil vs. BMI |
| 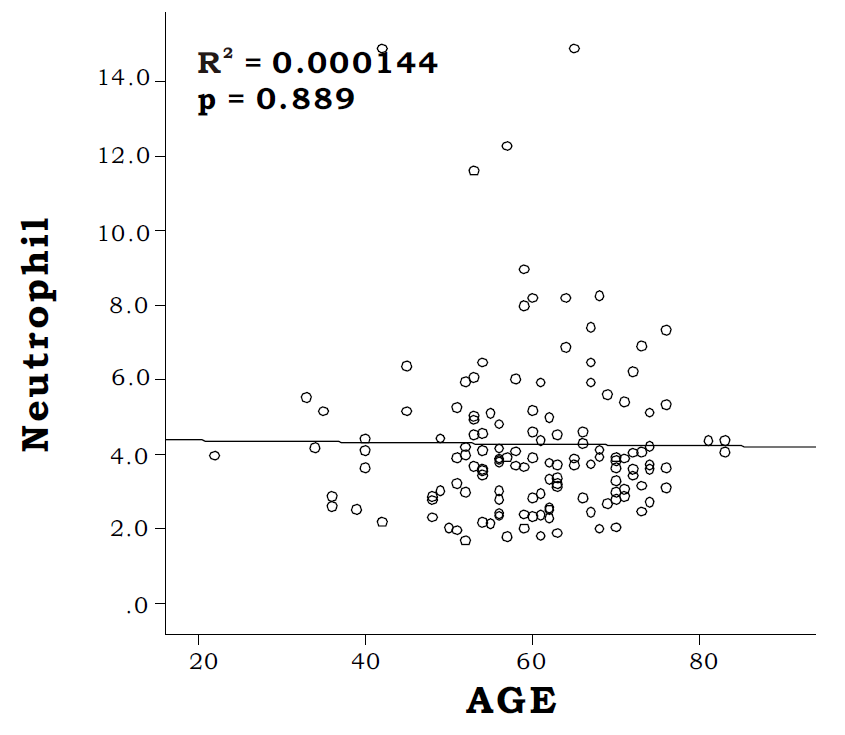 | 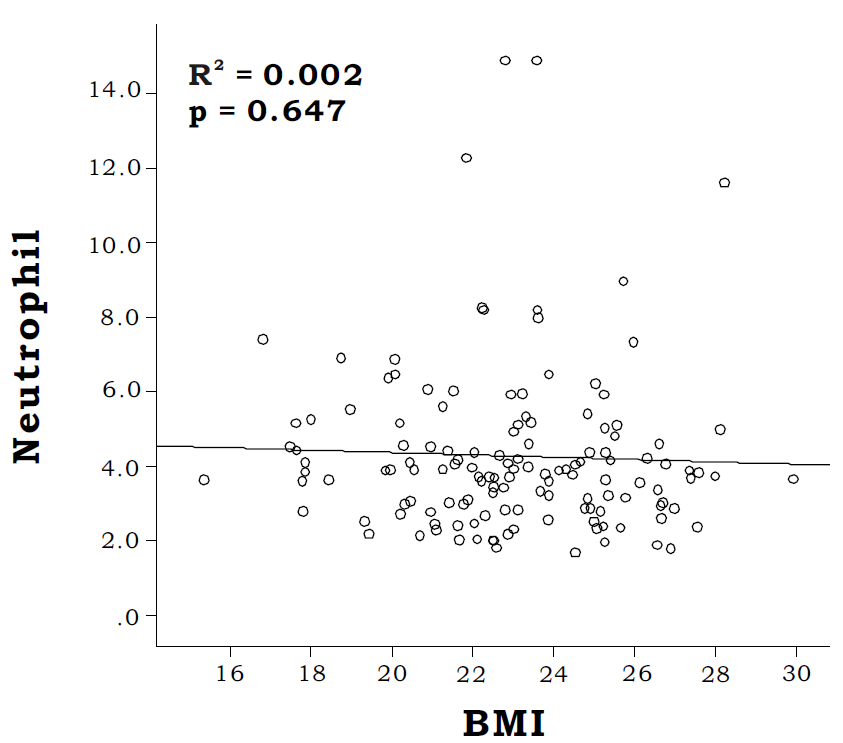 |
| E) Lymphocyte vs. Age | F) Lymphocyte vs. BMI |
| 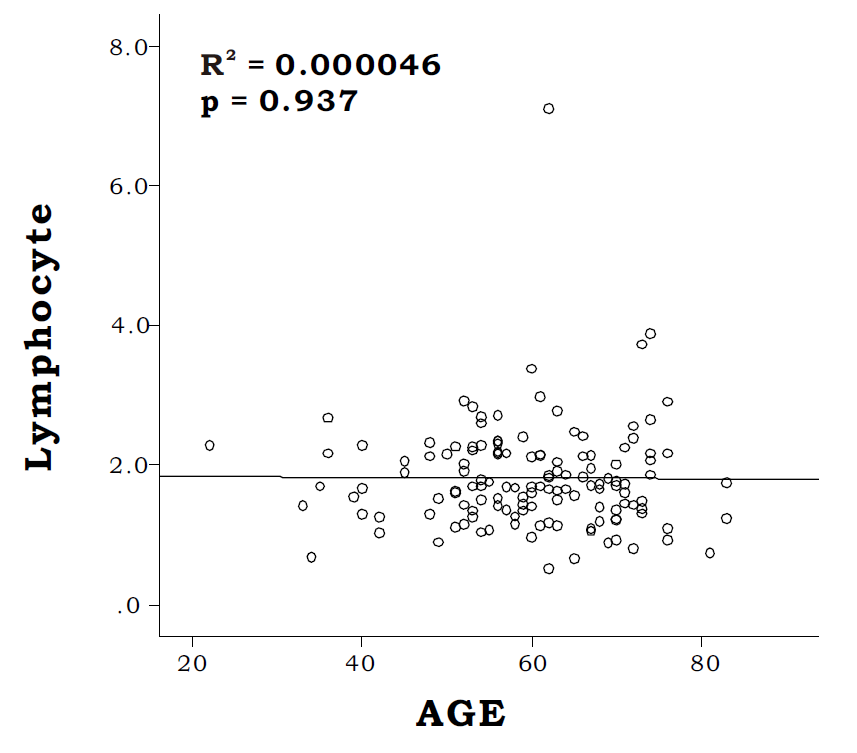 | 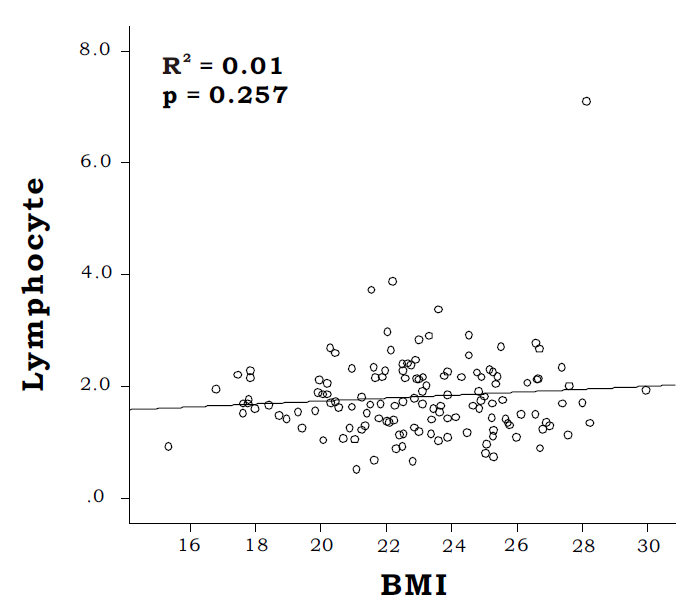 |
